# Development of a robot-assisted reduction and rehabilitation system for distal radius fractures

**DOI:** 10.3389/fbioe.2023.1342229

**Published:** 2024-01-10

**Authors:** Qing Zha, Zeou Xu, Hongbo Yang, Guodong Zhang, Xuefeng Cai, Wanlin Zhang, Yujiang Liu, Xiaofeng Shen, Yuwei Li

**Affiliations:** ^1^ School of Biomedical Engineering (Suzhou), Division of Life Sciences and Medicine, University of Science and Technology of China, Hefei, China; ^2^ Suzhou Institute of Biomedical Engineering and Technology, Chinese Academy of Science, Suzhou, China; ^3^ Suzhou TCM Hospital Affiliated to Nanjing University of Chinese Medicine, Suzhou, China

**Keywords:** distal radius fractures, robot-assisted, reduction, rehabilitation, biplane radiographic

## Abstract

**Background:** Closed reduction is the preferred treatment for distal radius fractures. However, it requires a multiple experienced medical staff and manually maintaining stable traction is difficult. Additionally, doctors cannot assess the reduction status of a fracture in real-time through radiographic images, which may lead to improper reduction. Furthermore, post-fracture complications such as joint adhesion, stiffness, and impaired mobility pose a challenge for the doctors. So it is necessary to optimize the treatment process of the distal radius fracture through technological means.

**Methods:** A robot-assisted closed reduction and rehabilitation system, which could assist doctors throughout the entire process of reduction, fixation, and rehabilitation of distal radius fractures, was developed. A mechanical system, composed of two grippers and a cooperative robotic arm, was used to grasp and tract the affected limb. A doctor controlled the robot through a joystick console and Windows application program. A biplane radiographic device was integrated into the system, which is not only convenient for doctors to view radiographic images of the fracture at any time but also for them to select the rotation axis of the wrist on the images before reduction and rehabilitation. Important information including the anteroposterior and lateral radiographic data and force and position parameters during the reduction and rehabilitation process were displayed on a graphic user interface.

**Results:** Experimental results showed that the proposed robotic system can meet the technical requirements for the reduction and rehabilitation of distal radius fractures, all the rotation angles could be achieved, a maximum force of more than 50 N could be achieved in all traction directions, and the error in selecting the wrist joint rotation axis line using radiographic images was less than 5 mm.

**Conclusion:** The developed robot-assisted system was shown to be suitable for closed reduction and rehabilitation of distal radius fractures, contributing a potential improvement in the quality of the procedures.

## 1 Introduction

Reduction, fixation, and rehabilitation are the three basic processes of fracture treatment. Reduction and fixation are the core treatments, and rehabilitation guarantees satisfactory functioning and efficacy of the limb after fracture surgery (Lichtman et al., 2011). Distal radius fracture is common in clinical settings. Displaced fractures are usually reduced using closed reduction methods, which are non-surgical and generally comprise traction and manipulation. The resulting position is then stabilized, typically by plaster cast immobilization (Handoll et al., 1996). The plaster cast immobilizes the wrist in a flexed, pronated, and ulnar deviated position for up to 6 weeks, often resulting in wrist pain and stiffness, especially during supination and extension (Charnley, 2003). Exercise is prescribed for at least 90% of patients after a distal radius fracture (Handoll et al., 2003). Physical therapy of joints, following surgery, focuses on both passive motion to restore mobility and active exercises to restore strength. Although a therapist perform passive motion for patients, continuous passive motion (CPM) devices have also been used. CPM improves recovery by stimulating the healing of articular tissues and circulation of synovial fluid, reducing local edema, and preventing adhesions, joint stiffness or contractures, or cartilage degeneration (Shirzadi et al., 2020). Adding mobilization with movement (MWM) to exercise and advice gives a faster and greater improvement in motion impairments for non-operative management of distal radius fracture (Reid et al., 2020). Thus Multiple experienced medical professionals are involved in the reduction, fixation, and rehabilitation of distal radius fractures. To overcome the drawbacks of traditional fracture reduction surgery, robot-assisted fracture reduction (RAFR) aimed to bring benefits, such as improved accuracy, less invasiveness, less radiation, a short hospital stay and accelerated postoperative rehabilitation (Zhao et al., 2020; Westphal et al., 2008). Therefore, various types of assistive robots have been developed. These robots are structured according to the following: (a) fixed external frame structure, (b) serial structure (such as that of an industrial robot), (c) parallel structure, and (d) serial-parallel hybrid structure (Bai et al., 2019). Machinery has surpassed human hands in terms of accuracy, stability, and repeatability. In fracture management, it can measure the angles, displacement, and force necessary for fracture reduction with precision and achieve perfect alignment of the fracture ends. Utilizing programmed fracture reduction treatment processes results in stable and consistent outcomes, reducing variations in results among doctors with different experience levels and years of service. In orthopedic surgery, robots have been developing rapidly in the past decades and are significantly beneficial to patients and healthcare providers (Zhao et al., 2020).

Regarding fracture reduction robots, research has primarily focused on the long bones of the limbs. Li et al. conducted a preliminarily study of a master and slave remote-controlled robotic system, and the experimental results showed high accuracy for fracture reduction and excellent performance (Li et al., 2016). Alruwaili et al. proposed a Wide-Open 3-armed parallel robot, Robossis, which can reach the boundary points of the workspace with a submillimeter accuracy and provide the required traction forces of up to 432 N to align femur fractures (Alruwaili et al., 2022). Zhu et al. designed and kinematically analyzed a femoral fracture reduction robot, which comprises a six-degree-of-freedom serial-link robot with three prismatic and three rotational joints. The proposed system has the potential for practical application in orthopedic clinical surgery (Zhu et al., 2022). Dagnino et al. designed a six-degree-of-freedom parallel robotic system for fracture manipulation, which allows for remote control in automatic mode and intra-operative adaptation for better reduction accuracy (root mean square error of 1.18 ± 1.14 mm [translations] and 1.85° ± 1.54° [rotations]) (Dagnino et al., 2015). Seide et al. developed a six-degree-of-freedom external fixator based on a hexapod robot, which had high-precision three-dimensional bone movement and could be expanded into a “smart fixator” in the future to automate controlled fracture and deformity treatment (Seide et al., 2004). Westphal et al. developed a robotic system for the reduction of femoral shaft fractures by utilizing modern techniques such as three-dimensional (3D) imaging, navigation, and robotics to overcome the disadvantages of the minimally invasive technique of intramedullary nailing, including malaligned fracture reductions and high radiographic exposure. The authors showed that high reduction accuracies could be achieved with the robotic system and that robot-assisted drill guidance achieves superior results compared with that achieved with the conventional procedure (Westphal et al., 2009). Priya et al. developed a novel method for reducing distal radius fractures using a mechanical device, which decreases the number of surgeons and time required to reduce the distal radius fracture and seeks to improve the accuracy of reduction (Priya et al., 2019). Xie et al. developed a novel fracture reduction device which enables only one doctor to complete the traditional manual reduction easily with precise measurements of all the necessary biomechanics and related parameters (Xie et al., 2016).

Regarding fracture rehabilitation robots, Picelli et al. supported the hypothesis that robot-assisted arm training might be a feasible tool for treating upper limb impairment in adult patients with distal radius fracture treated conservatively or surgically. The treatment of arm impairment consequent to distal radius fractures by means of robot-assisted arm training may allow therapists to focus on functional rehabilitation during occupational (individual) therapy and supervise (more than one) patients simultaneously during robotic training sessions (Picelli et al., 2020). César et al. designed and analyzed a horizontal rehabilitation robot based on a parallel mechanism for the treatment of femoral shaft fractures. Their designed robot helped patients to perform passive exercises of the hip. The system consists of three degrees of freedom actuated with linear actuators (Valdivia et al., 2013). Viriyasaranon designed and built a robot for elbow rehabilitation after elbow fractures, which could measure the limited range of motion of passive and active movements, measure stiffness of the human arm for passive movement, and provide assistive and resistive rehabilitation (Viriyasaranon, 2017). Wang et al. designed and implemented a soft parallel robot for automated wrist rehabilitation, which can assist the wrist to achieve all the required training motions, including abduction-adduction, flexion-extension, and supination-pronation motions (Wang and Xu, 2021). Noviyanto et al. designed a Continuous Passive Motion (CPM) machine for wrist joint therapy to reduce joint stiffness and improve joint mobility after surgery. The machine allows flexion, extension, ulnar, and radial movements of the wrist joint, with adjustable angles and speeds. The testing of the device showed a maximum difference of movement of 2°and a difference in speed of rotation of 0.5 s. The results indicate that the machine can be controlled according to the desired movement settings (Noviyanto et al., 2021). Kleber et al. integrated robotics and electronic games with the objective of producing more motivating and attractive therapeutic activities in distal radius fracture rehabilitation (wrist region) (Andrade et al., 2010). Cao et al. proposed innovative methods for circuit improvement, damping settings, and energy harvesting for rehabilitation training robots (Cao et al., 2022; Cao et al., 2023).

Closed reduction is the preferred treatment for distal radius fractures, but it requires the participation of multiple healthcare personnel, and maintaining stable traction manually through closed reduction is difficult. Additionally, doctors cannot assess the reduction status of the fracture on radiographic images in real-time, which may lead to improper reduction. Furthermore, post-fracture complications such as joint adhesion, stiffness, and impaired mobility are challenging for doctors. To address these issues, a robot-assisted closed reduction and rehabilitation system, which could assist doctors in completing the entire reduction, fixation, and rehabilitation process for distal radius fractures, was developed. The main contributions of this paper are as follows:

1) A mechanical system composed of two grippers and a cooperative robotic arm is used to grasp and provide traction to the affected limb. The doctor controls the robot through a joystick console and Windows application program.
2) A biplane radiographic device was integrated into the system, which is not only convenient for doctors to view the fracture on radiographic images at any time but also for selecting the rotation axis of the wrist based on the images obtained before reduction and rehabilitation.
3) Important information including the anteroposterior and lateral radiographic images and force and position parameters during the reduction and rehabilitation process were displayed on a graphic user interface (GUI).

## 2 Materials and methods

### 2.1 Clinical analysis

**FIGURE 1:**
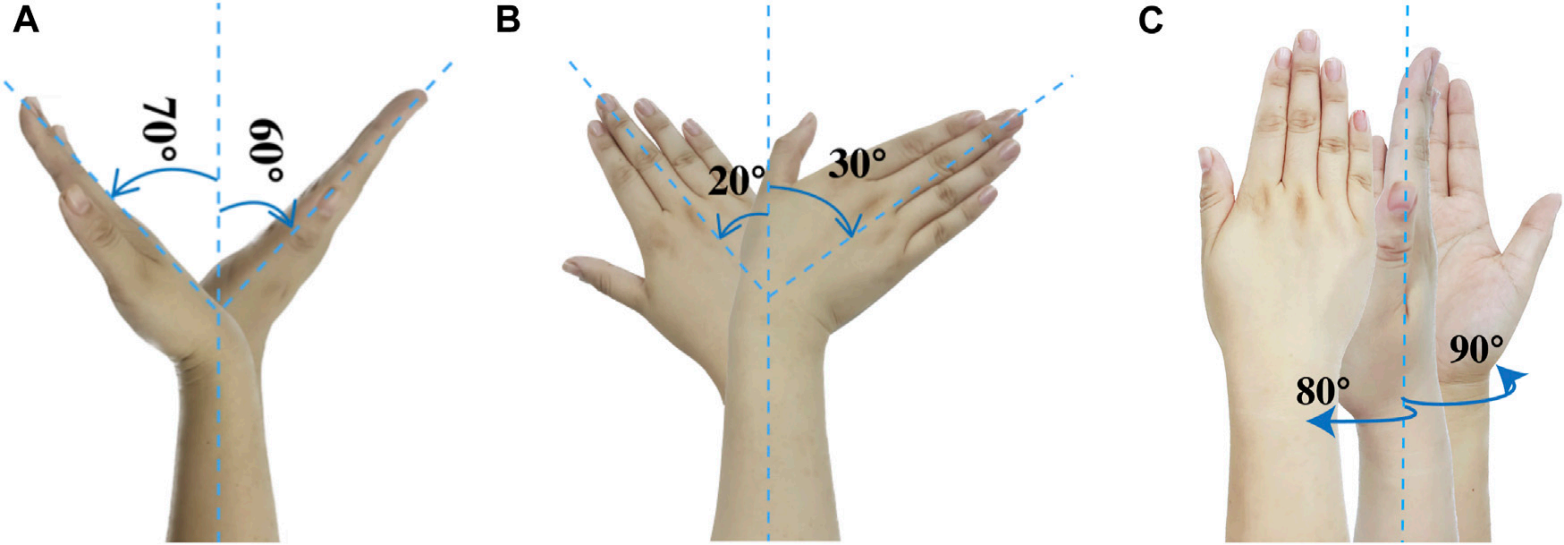
The angles of wrist movement. **(A)** Bending. **(B)** Tilting. **(C)** Rotating.

To achieve an effective design, it is essential to involve the inputs of primary and secondary stakeholders at the outset of the development process. Their knowledge and input aid in understanding present practices and identifying specific obstacles with current procedures and equipment (Georgilas et al., 2018). It is also important to develop a surgeon- and patient-friendly orthopedic surgical robot by imitating surgeons’ manual conduct of fracture reduction surgery and by maintaining surgeons’ way of thinking and planning surgeries (Zhu et al., 2021). The fracture reduction assistant robot is a novel medical equipment aimed to assist doctors to complete fracture reduction and rehabilitation more efficiently and accurately. To ensure the robot’s effectiveness and safety, it is crucial to design it based on clinical needs that meet the requirements of doctors. Therefore, we conducted a survey of 30 experienced doctors in the Bone and Joint Department of the Suzhou Hospital of Traditional Chinese Medicine who had more than 5 years of experience in distal radius fracture reduction. We gathered feedback and recommendations from different perspectives, and based on the survey results, we summarized the following required technical parameters:

1) Position of the patient’s body: Sitting or lying position.
2) Position of the affected limb: Shoulder abduction, 60°–90°; elbow flexion, 90° or 180°; and forearm and wrist in pronation or the neutral position.
3) Position for holding: The distal end is the palm or fingers, and the proximal end is the part of the forearm closer to the elbow joint.
4) Parameters of traction: Along the longitudinal axis of the limb, the traction force is controlled at 40–50 N depending on the specific situation. The traction time is preferably 1–3 min, and the traction displacement is about 5–10 mm. The range of wrist flexion and deflection angles is ±60° and ±30°, respectively
5) Continuous passive motion (CPM): The vertical bending wrist movement known as extension and flexion is shown in Figure 1A. The side-to-side horizontal tilting movement of the wrist, known as radial and ulnar deviation, is shown in Figure 1B. The wrist and forearm rotation movement, known as pronation and supination, is shown in Figure 1C.
6) Mobilization with movement (MWM): Anterior and posterior gliding, clockwise and counterclockwise rotation, longitudinal separation traction, and compression along the palmar axis of the wrist joint. Each action is performed in the functional position, which is palm flexion, ulnar deviation, wrist extension, and end-range radial deviation.

### 2.2 System configuration

**FIGURE 2:**
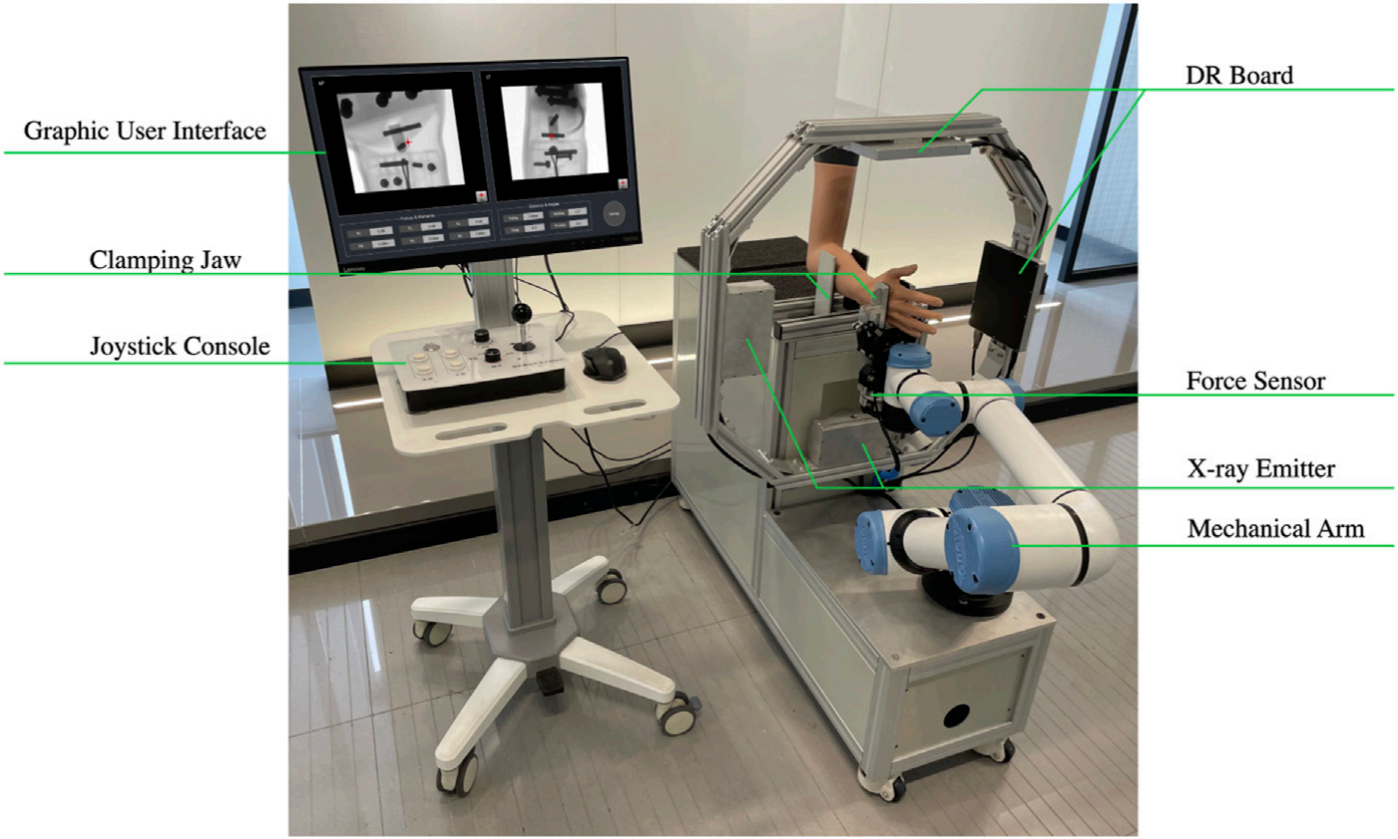
The robot-assisted reduction and rehabilitation system for distal radius fractures.

The robot-assisted closed reduction and rehabilitation system for distal radius fractures developed in this article is composed of the following elements: the robot body, lead protective curtain, mobile lead screen (Suzhou Kangshidun Protective Technology Co., Ltd., China), computer host (Advantech Co. Ltd., China), control console, and mobile cart as shown in Figure 2. The robot body consists of a movable base, a collaborative robotic arm (Aubo Intelligent Technology Co., Ltd., China), two sets of radiographic devices (Shanghai Anzhu Optoelectronic Technology Co., Ltd., China), and two gripping jaws (Shenzhen Dahuan Robot Technology Co., Ltd., China) and their parameters are shown in Table 1. The interaction between the doctor, robot, and patient forms a human-machine system, emphasizing the safety of medical personnel and patients in the design. Consequently, emergency stop buttons are present on both the robot body and control console. To ensure protection against radiations from radiography, a specially designed low-dose radiography machine is used for the forearm, effectively minimizing radiation exposure. The doctor is protected by a glass lead screen while the patient is shielded by a movable lead curtain, exposing only the affected limb to the radiation field. The collaborative robotic arm features collision detection and automatically stops when obstacles are encountered. It is equipped with a six-axis force sensor at its end, and the arm and palm clamps offer adjustable gripping force on the limb within an acceptable range. The control system monitors force in real time, and the robotic arm pauses automatically if the force exceeds the limit. The host serves as a relay station for all data and control transmission. The mobile lead curtain and lead screen provide protection for the patient and doctor, respectively. The upper part of the lead screen is a lift-up glass lead screen, enabling the doctor to easily observe the status of the robot and patient. The mobile cart facilitates close-range and long-range operations for the doctor.

**TABLE 1:** The parameters of robot-assisted reduction and rehabilitation system.

Parameter	Value
Collaborative robotic arm(AUBO i5)	d1 = 140.5 mm
	a2 = 408 mm
	a3 = 376 mm
Joint dimensions	d4 = 102.5 mm
	d5 = 102.5 mm
	d6 = 94 mm
Repeatability	±0.02 mm
Workspace (spherical)	886.5 mm (radius)
Load capacity	50 N
Clamping jaw for palm(DAHUAN AG95)	
Travel distance	0–95 mm
Clamping force	45–160N
Clamping jaw for palm(DAHUAN PGI-140)	
Travel distance	0–95 mm
Clamping force	40–140N
Six-axis force sensor(KUNWEI KWR75D)	
Force	Fx (500N), Fy (700N), Fz (700N)
Moment	Mx (18Nm), My (18Nm), Mz (18Nm)

### 2.3 Joystick console

**FIGURE 3:**
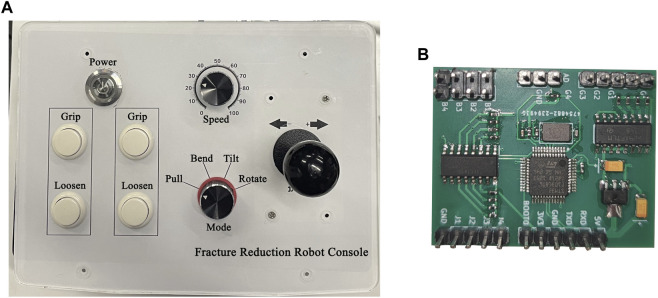
The joystick console. **(A)** Operation interface. **(B)** Circuit board.

**FIGURE 4:**
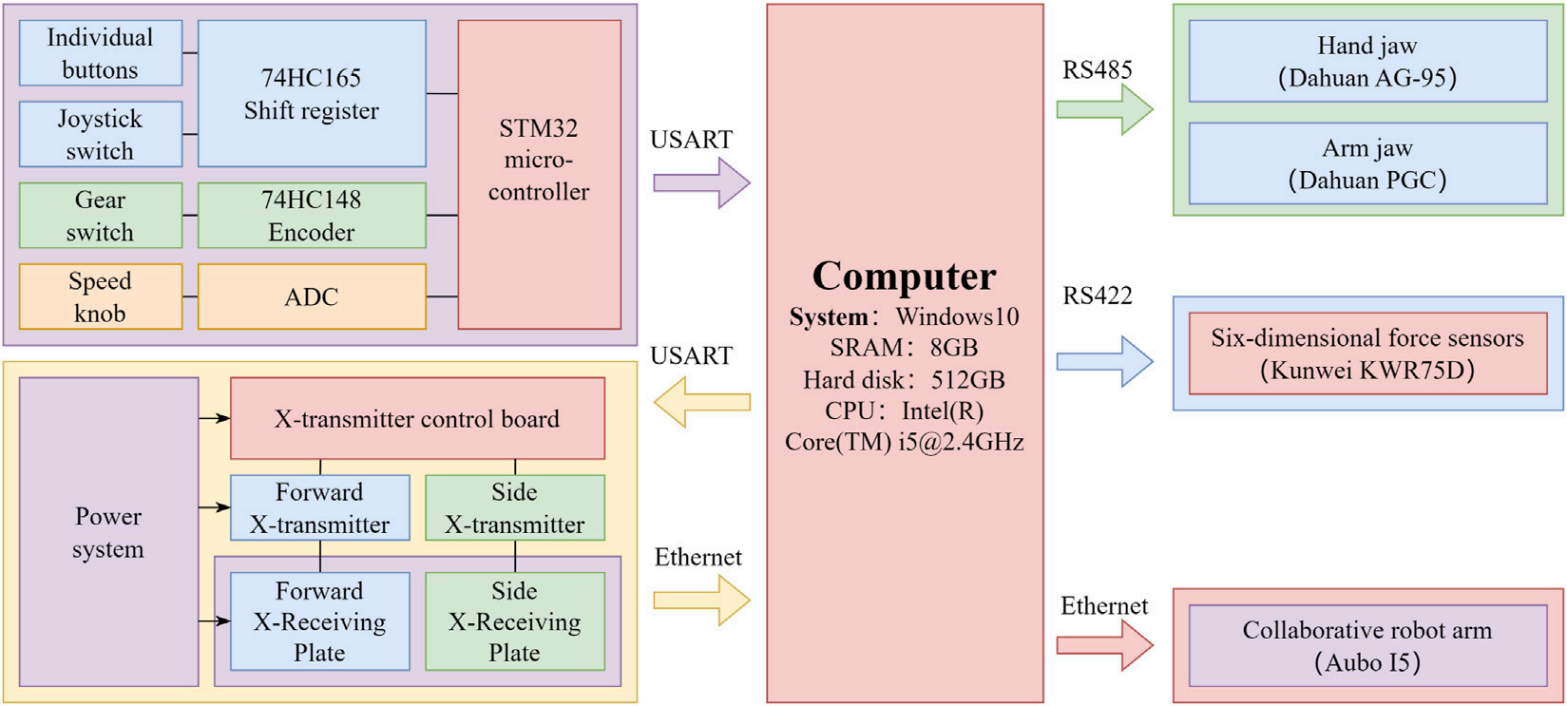
The block diagram of the control system.

The console’s main functions are to control the opening and closing of arm and palm grippers, adjust the movement speed of the robotic arm, select traction mode (drawing, palmar flexion, dorsal extension, ulnar deviation, and rotation), and implement movement. It controls the arm and palm grippers and adjusts the robotic arm’s movement through the host. As shown in Figure 3A, the console has four independent buttons including a four-speed switch, a four-direction joystick switch, and one knob. These components are abstracted into four independent modules that are connected to the main control module. The main control module, shown in Figure 3B, communicates with the upper computer and is based on STM32, the only microcontroller. As shown in Figure 4, the control module exchanges data with the upper computer through the USART serial port. After the upper computer sends specific instructions, the microcontroller returns a byte stream that includes status information of the four console components, such as whether all buttons are pressed, which gear position the switch is in, whether the joystick is being operated, and the position of the knob.

### 2.4 Biplane radiographic image acquisition system

**FIGURE 5:**
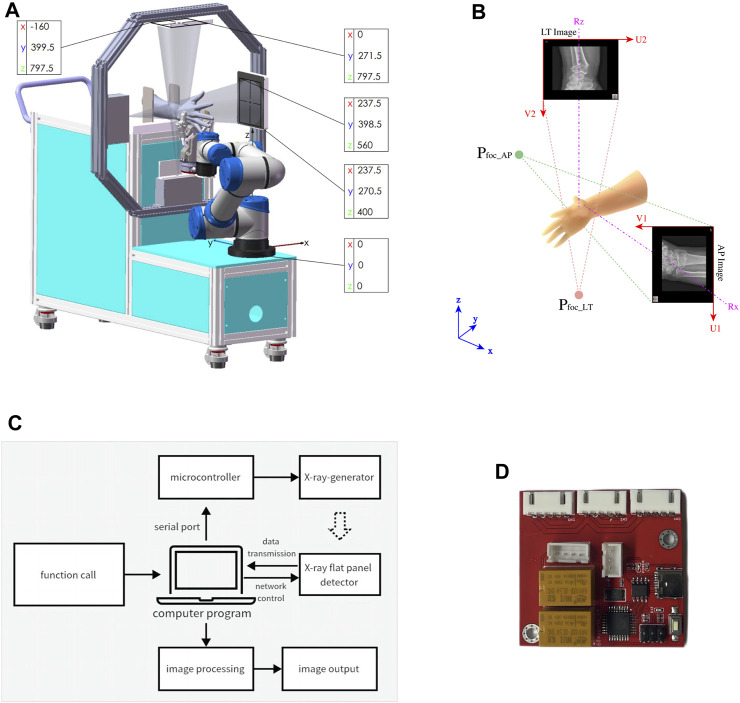
The biplane X-ray image acquisition system. **(A)** Block diagram. **(B)** circuit board. **(C)** Size and position. **(D)** Coordinates of image.

The large G-arm radiography machines employed in hospitals are costly and unwieldy, which are excessive for distal radius fractures. As shown in Figures 5A, B, by integrating radiography machines into robots, the positional relationship between the limbs in the image and the actual physical space can be determined. As shown in Figures 5C, D, the biplane radiographic image acquisition system comprises three main components including radiography source control, reception panel network layer control, and primary image processing. The base coordinate origin of the robotic system is set at the center of the flange at the base of the collaborating robotic arm. The detection area of the receiving plate is 160 mm × 128 mm, and the pixels of the radiographic images are 640 × 512. As shown in Figure 4, the computer host connects to the microcontroller and two radiography receiving panels through a USB serial port and two RJ45 network interfaces, respectively. The system controls two sets of radiographic emission-reception devices separately in the vertical and horizontal directions based on the set exposure parameters. After image processing, the acquired radiographic images are displayed. A doctor can activate the acquisition of anteroposterior and lateral radiographic images using a foot pedal.

### 2.5 Graphic user interface

**FIGURE 6:**
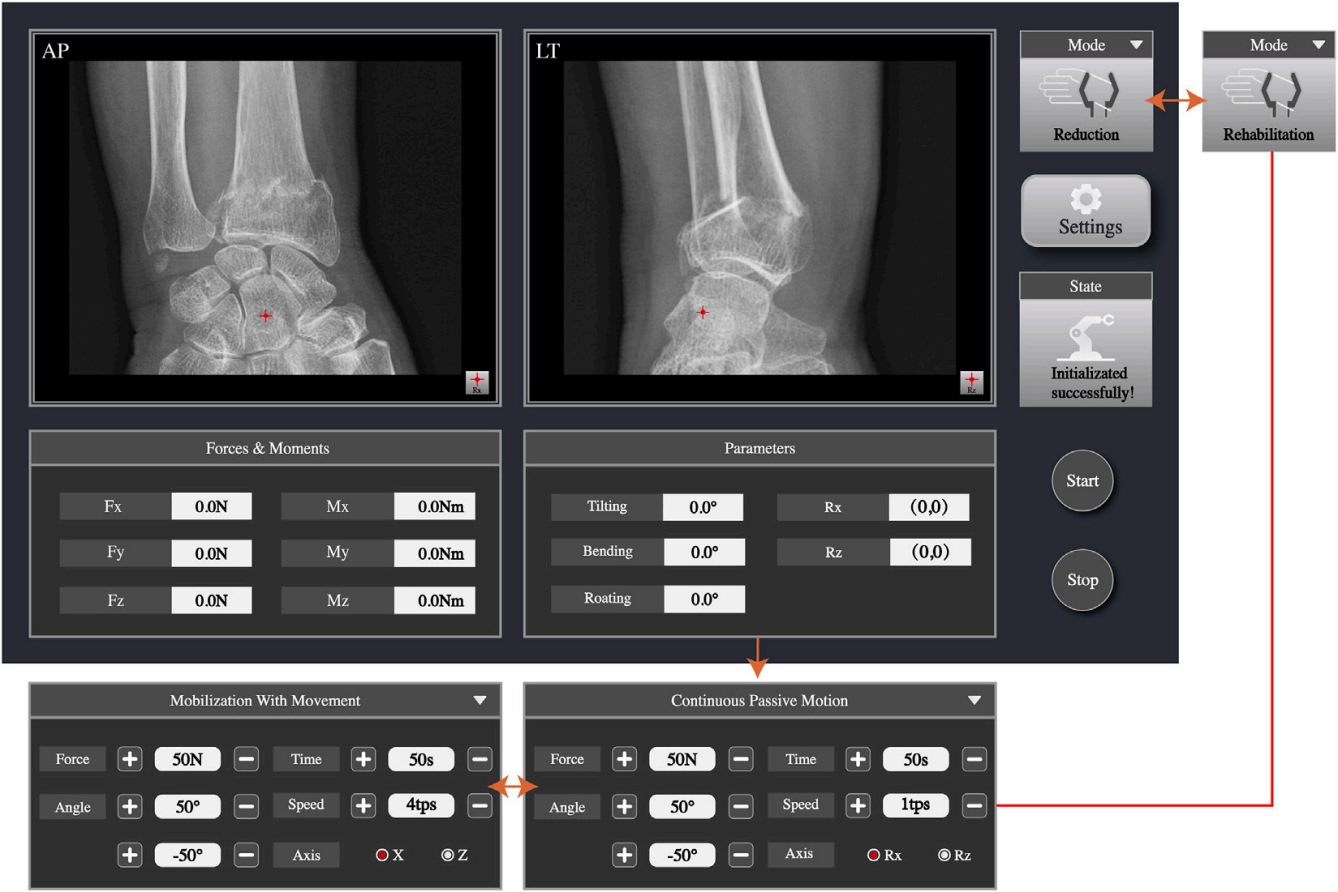
The Graphic User Interface (GUI) of the system.

The GUI is used to display the images and parameter data the doctor needs to view during fracture reduction. Meanwhile, in rehabilitation training mode, the doctor can set rehabilitation training parameters and start or stop the training through the GUI. As shown in Figure 6, the anteroposterior and lateral images can be displayed simultaneously, enabling doctors to check the fracture situation in real time. The doctor can set the rotation axis of the end of the robotic arm based on the anteroposterior and lateral radiographic images. The velocity of the robotic arm and the gripping force of the jaws can also be adjusted. The gripping force can be adjusted to 40–50 N for individuals with less muscles and can reach up to 140 N for those with more muscles. The maximum pressure a human body can withstand is 140 N, so the gripper will not cause harm to the body. Furthermore, the six-dimensional force sensor collects data parameters of force and moment, which are displayed on the interface. After the rotation axis is set, the traction force and position parameters are reset to zero. During reduction, parameters of force and position for pulling, bending, tilting, and rotating in real time are displayed on the software interface and parameters such as traction force, speed, range, time, and axis of rotation can be set. The connection status of the robot and radiography machine is displayed on the interface.

### 2.6 Force and torque compensation

To obtain the force and torque of the robot on the affected limb in real time, a six-dimensional force sensor was installed under the clamp claw. Due to the low-speed robotic movements, the influence of inertial force can be neglected. Force sensor data fluctuations are ignored and the end tooling is not replaced. Therefore, the load is subject to external contact force and only needs influence of a sensor system error, and load gravity action should be eliminated. By selecting the six-dimensional force sensor at different poses, the gravity size and the center of gravity position of the load end can be calculated (Kim et al., 2013).

**FIGURE 7:**
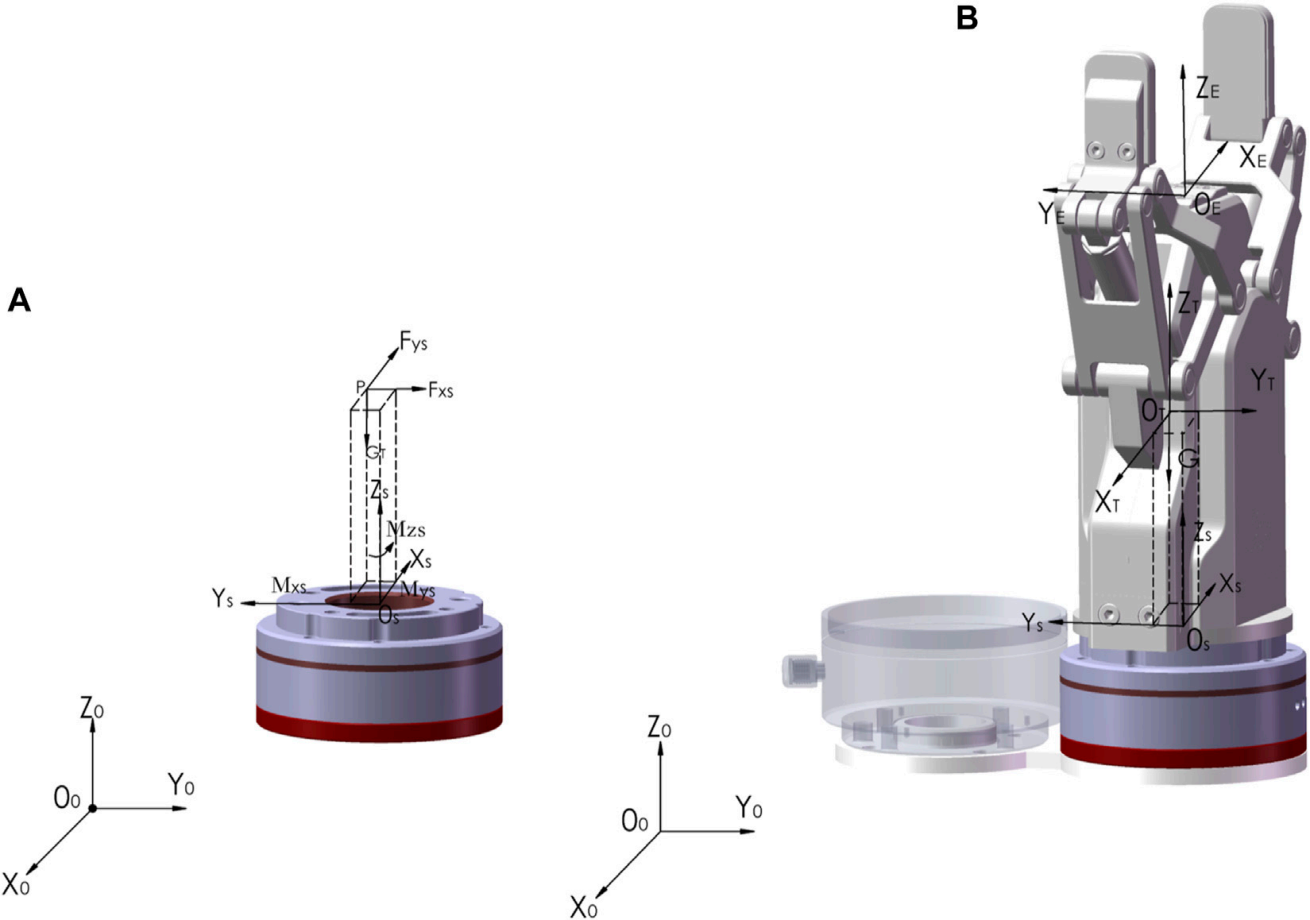
The force and torque calibration of six-dimensional force sensor. **(A)** Gravity and center of mass. **(B)** The coordinate systems.

As shown in Figure 7A, the coordinate systems are established, and the mechanical arm base coordinate system is $O_{O}-X_{O}Y_{O}Z_{O}$ coordinate system { O }. The measurement coordinate system of the six-dimensional force sensor is ${\;O}_{S}-X_{S}Y_{S}Z_{S}$ , as coordinate system { S }. The coordinates of the center of mass ( $p_{\text{xS}},p_{\text{yS}},p_{\text{zS}}$ ), the zero value of the force components $\left(F_{x0},F_{y0},F_{z0}\right),$ and the zero value of the torque components $\left(M_{x0},M_{y0},M_{z0}\right)$ in the six-dimensional force sensor coordinate system can be calculated from multiple sets of data using the least-squares method by the following equation.

$$\left[\begin{array}{c} M_{\text{xS}}\\ M_{\text{yS}}\\ M_{\text{zS}} \end{array}\right]=\left[\begin{array}{cccccc} 0 & F_{\text{zS}} & -F_{\text{yS}} & 1 & 0 & 0\\ -F_{\text{zS}} & 0 & F_{\text{xS}} & 0 & 1 & 0\\ F_{\text{yS}} & -F_{\text{xS}} & 0 & 0 & 0 & 1 \end{array}\right]\left[\begin{array}{c} p_{\text{xS}}\\ p_{\text{yS}}\\ p_{\text{zS}}\\ k1\\ k2\\ k3 \end{array}\right]$$

where $\left\{\begin{array}{c} k_{1}=M_{x0}+F_{y0}\times p_{\text{zS}}-F_{z0}\times p_{\text{yS}}\\ k_{2}=M_{y0}+F_{z0}\times p_{\text{xS}}-F_{x0}\times p_{\text{zS}}\\ k_{3}=M_{z0}+F_{x0}\times p_{\text{yS}}-F_{y0}\times p_{\text{xS}} \end{array}\right.$

Then load-end gravity $G_{T}=\sqrt{F_{xS}^{2}+F_{yS}^{2}+F_{zS}^{2}}\;$

After the load-related parameters are determined, as shown in Figure 7B, the coordinate system { T } at the center of gravity point, which is $O_{T}-X_{T}Y_{T}Z_{T}$ , is established. The direction of { T }is the same as that at the base coordinate system { O }. The coordinate system is established at the action point of the mechanical arm and the external force, $O_{E}-X_{E}Y_{E}Z_{E}$ , as { E }. The direction of { E } is the same as that of the coordinate system { S }. Map the gravity and torque of the load end under the coordinate system {𝑇} to the coordinate system {𝑆}, and the calculation formula of the gravity and torque value that can represent the load end compensation of the sensor is

FSCMSC=RST0lPTSRSTRSTFTMT

where load-end gravity and torque is ${\left[F_{T}\;M_{T}\right]}^{T}={\left[0\;0\;-\left|G_{T}\right|\;0\;0\;0\right]}^{T}\;$ , lPTS=0−pzSpySpzS0−pxS−pySpxS0 is operator matrix , ${\;}_{S}^{T}R=\left[\begin{array}{ccc} -1 & 0 & 0\\ 0 & -1 & 0\\ 0 & 0 & 1 \end{array}\right]\text{ is}$ the rotation transformation matrix of the coordinate system {*T*} relative to the coordinate system {*S*}.

Finally the external force on the end of the robotic arm can be calculated by the following equation.

FEME= ESR0lPES ESR ESR−1FSMS−FSCMSC−F0M0

In which lPES=0−pzEpyEpzE0−pxE−pyEpxE0 is operator matrix, ESR=100010001 is the rotation transformation matrix of the coordinate system {S} relative to the coordinate system {E}.

## 3 Results

### 3.1 Axis alignment error

The biplane radiograph system is mounted and calibrated according to the parameters of the robotic system, and we use a metric ruler with a square-handled ball and an embedded lead scale to mount, calibrate, and measure error. Sources of error include the aluminum alloy profile material dimensions, mounting, center emission point of the radiographic emitter, and the receiving plate. The flat plate received rays that are similar to the radiograph emission angle of ±19° and greater than the detector receiving angle of 16.3°. The arm could be completely imaged on the receiving plate. According to the principle of radiography, the magnification factor (Magnification Factor) can be calculated by Mf=d/D where d is the distance between object and emission source, D is the distance between emission source and receiving plate. We conducted a positioning accuracy experiment using a 3D printed spherical ball with a handle and the transfer plate on the robotic arm. Three different positions were selected in both the anterior and lateral directions for X-ray imaging, then center points were selected on the acquired images to test positioning accuracy. Table 2 presents the test results, indicating that the coordinate error of the spherical center remains within a 5 mm range.

**TABLE 2:** Positioning accuracy from image to space.

Object	Error (mm)					
	Vertical (x,y)			Horizontal (y,z)		
	P1	P2	P3	P1	P2	P3
Sphere	(0.65,2.13)	(0.86,2.87)	(1.06,3.34)	(0.94,2.69)	(1.35,3.47)	(1.91,4.28)
Disk	(0.74,1,85)	(0.93,1.80)	(1.42,3.74)	(0.36,0.50)	(0.64,0.44)	(1.52,0.40)

### 3.2 Traction parameters

According to Section 2.1, during the fracture reduction and rehabilitation, the robotic arm needs to exert the maximum traction force between 40 and 50 N in each required position. We measured the angle at the end of the robotic arm through the digital display inclinometer (TLL-90S, Dongguan Jingyan Instrument Technology Co., LTD., China) and assessed whether the traction force with the pull pressure gauge (SSMCL-YL-1kN, Shenzhen You Zhongli Technology Co., LTD., China) meets the requirements. The traction force of the robotic arm along the axis of the arm can reach more than 50 N at different angles.

### 3.3 Simulated reduction

Using the fracture reduction-assisted robot developed in this study, we requested chief physicians from the Suzhou Hospital of Traditional Chinese Medicine to test the auxiliary reduction and rehabilitation training function with a Colles' Fracture Reduction Trainer. The reduction steps are as follows: (1) connect the power supply, open the collaboration mechanical arm control cabinet, start the master control computer, and initialize; (2) place the hand model in the appropriate position on the machine and hold it with the clip claw; (3) step on the radiography machine pedal to obtain the forward side image, observe the fracture situation and select the wrist joint axis on the image; (3) the doctor operates the robotic arm along the axis of the arm through the rocker arm console and positions the robot in the palm flexion deviation; (4) the doctor adjusts the traction and angle during the manual reduction, and (5) adjusts the traction force and angle after the reduction; and (6) the clip claw is released after fixation, and the arm model is removed. The experimental results show that the robot can effectively hold the affected limb and implement the required traction when the doctor implements the manual reduction and external fixation of the fracture model.

### 3.4 Simulated rehabilitation

In the rehabilitation mode, the rehabilitation therapist should first control the mechanical arm through the joystick console to drive the movement of the affected limb to obtain the compression and traction forces a patient’s wrist joint can withstand and to obtain achievable training angles. The automatic movement of the mechanical arm is then observed through the software interface. First, the rehabilitation mode, CPM or MWM, is selected. Subsequently, the rotational axis of the wrist is selected on the lateral radiographic image; the traction force, direction, angle, speed, and frequency of joint movements are set, and the start button is pressed. When choosing CPM mode, the arm is held so it can only be achieved bending and tilting. Their axes of rotation were selected on the AP (Rz) and LT (Rx) X-ray images. When choosing MWM mode, the arm is in a semi-restricted position and the wrist motion are faster linear reciprocating motion in the 30 mm range of the XZ directions. Table 3 illustrates the results of experimental tests, indicating that both the robot-assisted CPM and MWM are capable of fulfilling the usage requirements of rehabilitation therapists.

**TABLE 3:** Parameters for reduction and rehabilitation of the robot.

Traction force	Reduction		Rehabilitation			
			CPM(0∼2tps)		MWM(0∼4tps)	
	Bending	Tilting	Rx	Rz	X (mm)	Z (mm)
0N ∼ 50N	−5°∼5°	−30°∼30°	−5°∼5°	−30°–30°	±30	±30
No load	−60°–70°	−30°∼30°	−30°∼20°	−60°–70°	±30	±30

## 4 Discussion

The development of the robot-assisted system for fracture reduction and rehabilitation is an innovative and potentially game-changing advancement in orthopedic surgery. This technology, which can be challenging and complex, aims to improve the accuracy, precision, and safety of the reduction and rehabilitation procedure. Therefore, all aspects of the robot including the form of the robotic arm (Lin et al., 2013; Wang et al., 2013; Zhu et al., 2017; Georgilas et al., 2019), the connection technology between the bone and the robot (Yang et al., 2021), the force and moment in the reduction process (Zhu et al., 2016; Lei et al., 2020), functional evaluation (Hung and Lee, 2010), accuracy evaluation (Li et al., 2014), and interaction mode (Suero et al., 2018), have been studied. By utilizing a robot, surgeons can enhance their surgical techniques by obtaining real-time imaging guidance, improving their visualization of the fracture site. The robot can assist with the precise manipulation and repositioning of the fractured bone fragments, ensuring optimal alignment and stability during the reduction process. One of the key benefits of using a robot for closed reduction is the potential for decreased tissue trauma and reduced surgical time. By relying on robot-assisted techniques, surgeons can minimize soft tissue damage and achieve more efficient surgeries, which may lead to the quicker recovery of patients. Additionally, the use of robotics in closed reduction procedures can potentially decrease the risk of complications, such as improper alignment or unstable fixation, which are common challenges of traditional manual reduction techniques. The robot’s ability to perform repetitive and precise movements may improve overall outcomes and enhance the quality of care provided to patients. However, it is important to note that the development of a robot for closed reduction and rehabilitation of distal radius fractures is still an ongoing area of research and development. The technology is not yet widely available or fully optimized for clinical use. Further studies and trials are necessary to assess its safety, efficacy, and cost-effectiveness before widespread implementation. Nevertheless, the potential benefits of a robot-assisted closed reduction and rehabilitation system for distal radius fractures hold promise for the future of orthopedic surgery, paving the way for advancements in surgical techniques and ultimately improving patient outcomes.

In this study, empirical parameters such as the position of the patient and affected limb, clamping position, direction of traction and reduction, angle, and force required for fracture reduction were obtained through clinical research, guiding the development of the distal radius fracture reduction and rehabilitation robot. The structure, hardware, and software of the reduction and rehabilitation robot were developed, and parameter indexes were tested. Experiments were conducted using a distal radius fracture model. The tests and results demonstrate that the developed closed reduction auxiliary robot for distal radius fractures can effectively assist doctors in completing the reduction, fixation, and rehabilitation process by enabling the binding of the affected limb, multi-degree-of-freedom traction, and real-time display of radiographic images.

The robot-assisted system for distal radius fractures can assist doctors in performing closed manual reduction and assist in rehabilitation. Lead screens and lead curtains are used to protect doctors and patients from radiation. The radiation emitted by small radiography machines is significantly smaller than that of large C-arm or G-arm radiography machines. The mechanical arm is operated using a joystick console to achieve fracture reduction, while the radiography machine only needs to be turned on during fracture analysis and viewing. Mostly, the machine remains in a non-radiation state, allowing doctors to view the images up close. With the help of the radiography device, doctors can monitor a patient’s fracture status in real time through the display screen, while maintaining the position of the patient’s arm using the robotic arm and gripper system, thereby achieving precise reduction. This eliminates the need for the patient to visit the radiology department multiple times before and after the operation, ultimately reducing medical costs.

The developed closed reduction and rehabilitation auxiliary robot system for distal radius fractures is suitable for outpatient orthopedics and traumatology clinics. It has low radiation dose and includes movable lead curtains and screens. The system is compact in size and easy to install. Patients can directly obtain radiographic images in the clinic using the system. After diagnosis by a doctor, if the patient meets the applicable fracture range of the system, the doctor proceeds with the reduction procedure after obtaining the patient’s consent. However, it can only achieve auxiliary traction and rehabilitation functions, cannot directly apply forces to the fractured bones for reduction purposes, such as the technique of manipulating the fracture site. It also lacks intelligent functions such as guidance based on X-ray images and force feedback control. The next step involves adding a top-folding mechanism to directly apply force to the broken bone to better simulate manual reduction, as well as incorporating functions such as artificial intelligence fracture classification and reduction guidance. Further experiments are necessary to verify the influence of muscle strength on various animal bones and cadavers, analyze the performance of the control system, and optimize the mechanism of the robotic system for patient safety and convenience before applying it to clinical environments in the future. Currently, there are no mature commercialized products for a robotic system for distal radius fractures worldwide, and many research institutes and hospitals are still in the exploratory stage of research about this system. Building an auxiliary fracture reduction robotic system based on medical image guidance to assist doctors in completing distal radius fracture reduction and achieving precise minimally invasive surgery hold great medical potential.

## Data Availability

The original contributions presented in the study are included in the article/Supplementary material, further inquiries can be directed to the corresponding authors.
